# Determination of complex subclonal structures of hematological malignancies by multiplexed genotyping of blood progenitor colonies

**DOI:** 10.1016/j.exphem.2017.09.011

**Published:** 2018-01

**Authors:** Francesca L. Nice, Charlie E. Massie, Thorsten Klampfl, Anthony R. Green

**Affiliations:** a Department of Haematology, Cambridge Institute for Medical Research University of Cambridge, Cambridge CB2 0XY, United Kingdom; b Wellcome Trust/Medical Research Council Stem Cell Institute, University of Cambridge, Cambridge CB2 1QR, United Kingdom; c Department of Haematology, Addenbrooke's Hospital, Cambridge CB2 0QQ, United Kingdom

## Abstract

Current next-generation sequencing (NGS) technologies allow unprecedented insights into the mutational profiles of tumors. Recent studies in myeloproliferative neoplasms have further demonstrated that, not only the mutational profile, but also the order in which these mutations are acquired is relevant for our understanding of the disease. Our ability to assign mutation order from NGS data alone is, however, limited. Here, we present a strategy of highly multiplexed genotyping of burst forming unit-erythroid colonies based on NGS results to assess subclonal tumor structure. This allowed for the generation of complex clonal hierarchies and determination of order of mutation acquisition far more accurately than was possible from NGS data alone.

## Highlights

• Complex clonal hierarchies are difficult to predict computationally from NGS data.
• Multiplexed genotyping of BFU-Es based on NGS data allowed for the determination of a complex subclonal composition in a patient with MPN.
• Analysis of subclonal composition allowed for the determination of the order of acquisition of mutations.

Next-generation sequencing (NGS) methods have provided unprecedented insights into the somatic mutations associated with hematological malignancies, including myeloproliferative neoplasms (MPNs) 1, 2, 3. However, although we are now able to acquire detailed lists of mutations present in tumors at a given state of development, we are only beginning to understand how these mutations are associated in tumor subclones and the history of mutation acquisition during tumor development. The relevance of analyzing the makeup of tumors in detail has been demonstrated recently in studies of MPN patients and have shown, for the first time in any cancer, that the order in which somatic mutations are acquired influences tumor biology and clinical presentation 4, 5.

However, determining the subclonal architecture of tumors accurately remains challenging. In particular, mutant allele burdens determined by NGS have a limited ability to predict the clonal landscape and clonal history within a patient. Clonal analysis of hematopoietic colonies provides a powerful approach to circumvent the problems associated with sequencing pooled cell populations 1, 2, 4, 5. Here, we present our strategy to determine complex subclonal tumor structures using a combination of NGS and highly multiplexed genotyping of burst forming unit-erythroid colonies (BFU-Es).

## Methods

### Clustering analysis

Clustering was carried out using MClust Version 3 for R [6] on the basis of NGS-derived mutational allele burden data for patient PD4772. MClust utilizes normal mixture modeling for univariate data to classify the allele burdens into clusters as a prediction for the subclonal structure of the tumor.

### Blood acquisition and processing

Patient PD4772 was recruited from Addenbrookes Hospital after written informed consent and ethical approval consistent with the Declaration of Helsinki. As described previously [4], mononuclear cells (MNCs) were isolated from 40 mL of peripheral blood using a sodium diatrizoate/polysaccharide density gradient (Lymphoprep; Axis Shield PLC, Oslo, Norway) according to the manufacturer's instructions. MNCs were plated at a density of 1 × 10^6^ cells/mL in MethoCult H4034 (StemCell Technologies, Vancouver, Canada). BFU-Es were identified and picked into 100 µL of PBS before vigorous pipetting to break the colony apart. Of the 100 µL cell suspension, 10 µL was used for capillary sequencing and 10 µL for Fluidigm SNP genotyping.

### Fluidigm SNP genotyping

Fluidigm SNP genotyping was performed according to the manufacturer's instructions (SNP Genotyping User Guide, PN 68000098 M2, Appendix C: SNP Type Assays for SNP Genotyping on the 192.24 Dynamic Array Integrated Fluidics Circuit, IFC). Briefly, SNPType genotyping assays were designed for all mutations identified previously in patient PD4772 by NGS according to the manufacturer's recommendations and ordered from Fluidigm (Supplementary Table E1, online only, available at www.exphem.org).

Predefined regions of DNA were amplified using polymerase chain reaction (PCR) with specific target amplification primers for 22 cycles before a 1:100 dilution of the amplified products was prepared. The diluted amplified product was loaded onto the 192.24 Dynamic Array IFC for SNP Genotyping (BMK-M-192.24GT, Fluidigm) alongside a sample premixture including ROX reference dye and real-time master mixture. Assays were composed of allele-specific primers tagged with either FAM or HEX and an untagged common locus-specific PCR primer. The array was processed using the BioMark system (Fluidigm), which performs the thermal cycling and image acquisition.

Data were analyzed using the Biomark SNP Genotyping Analysis software version 3.1.2 to obtain genotype calls. Briefly, the software calculates the relative fluorescence intensities of FAM and HEX compared with the background ROX signal, classifying each of the data points into one of three genotypes (wild-type, heterozygous mutant, or homozygous mutant) using *k-means* based clustering methods.

### Capillary sequencing

Capillary sequencing was carried out as described in Ortmann et al [4]. Sequences of the primers used in this study are provided in Supplementary Table E2 (online only, available from www.exphem.org).

## Results/discussion

**Figure 1:**
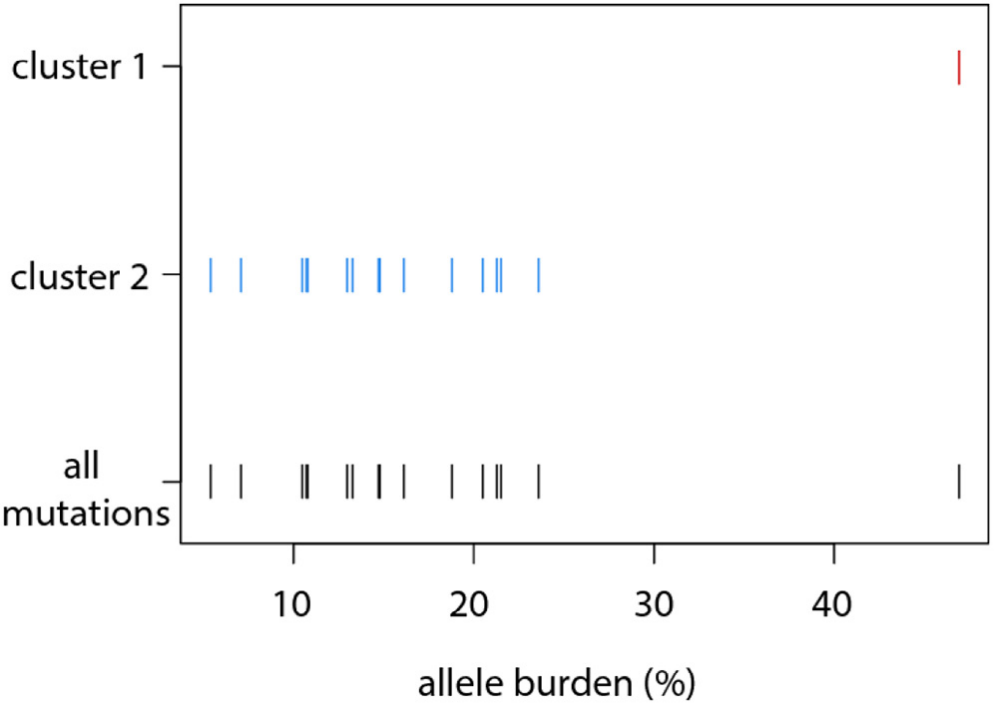
Prediction of clonal structure from mutant allele burden alone for patient PD4772. Predicted mutant allele burdens from whole-exome sequencing were visualized using the MClust classification plot. Each line represents a mutation shown both as part of the two identified clusters (red and blue) and combined (black). Cluster 1 is defined only by the JAK2^V617F^ mutation and all other mutations are found in cluster 2.

Whole-exome sequencing of bulk granulocyte DNA from polycythemia vera patient PD4772 revealed 16 somatic mutations with a range of mutant allele burdens (Supplementary Table E1, online only, available from www.exphem.org [1]). We set out to determine the subclonal tumor composition in this patient from a comparison of mutant allele burdens alone by means of a clustering analysis (Fig. 1). The result of this analysis revealed two separate clusters of mutations indicating the presence of two tumor subclones. The JAK2^V617F^ mutation had the highest allele burden (46.9%), defining cluster 1 (Fig. 1). The second cluster contained the remainder of all other mutations, where allele burdens ranged from 5.4% to 23.6% (cluster 2, Fig. 1). Although the algorithm was not able to determine further clusters given the wide range of mutant allele burdens within cluster 2, we hypothesized a more complex subclonal makeup of this tumor. Given the relatively low allele burdens, a potential serial acquisition of two mutations cannot be distinguished from a biclonal acquisition using analysis that is based on allele burden alone. Moreover, the analysis did not provide insights in the historical development of the tumor.

In order to gain a more comprehensive understanding of this patient's tumor, we analyzed 176 BFU-E colonies that were cultured from peripheral-blood-derived mononuclear cells. Each of these colonies originated from a single blood progenitor cell. Genotyping each colony therefore provides information of the genetic makeup of the tumor at single-cell resolution. The combined interpretation of genotypes from a large number of individual colonies allows conclusions to be drawn about the subclonal composition of the tumor.

**Figure 2:**
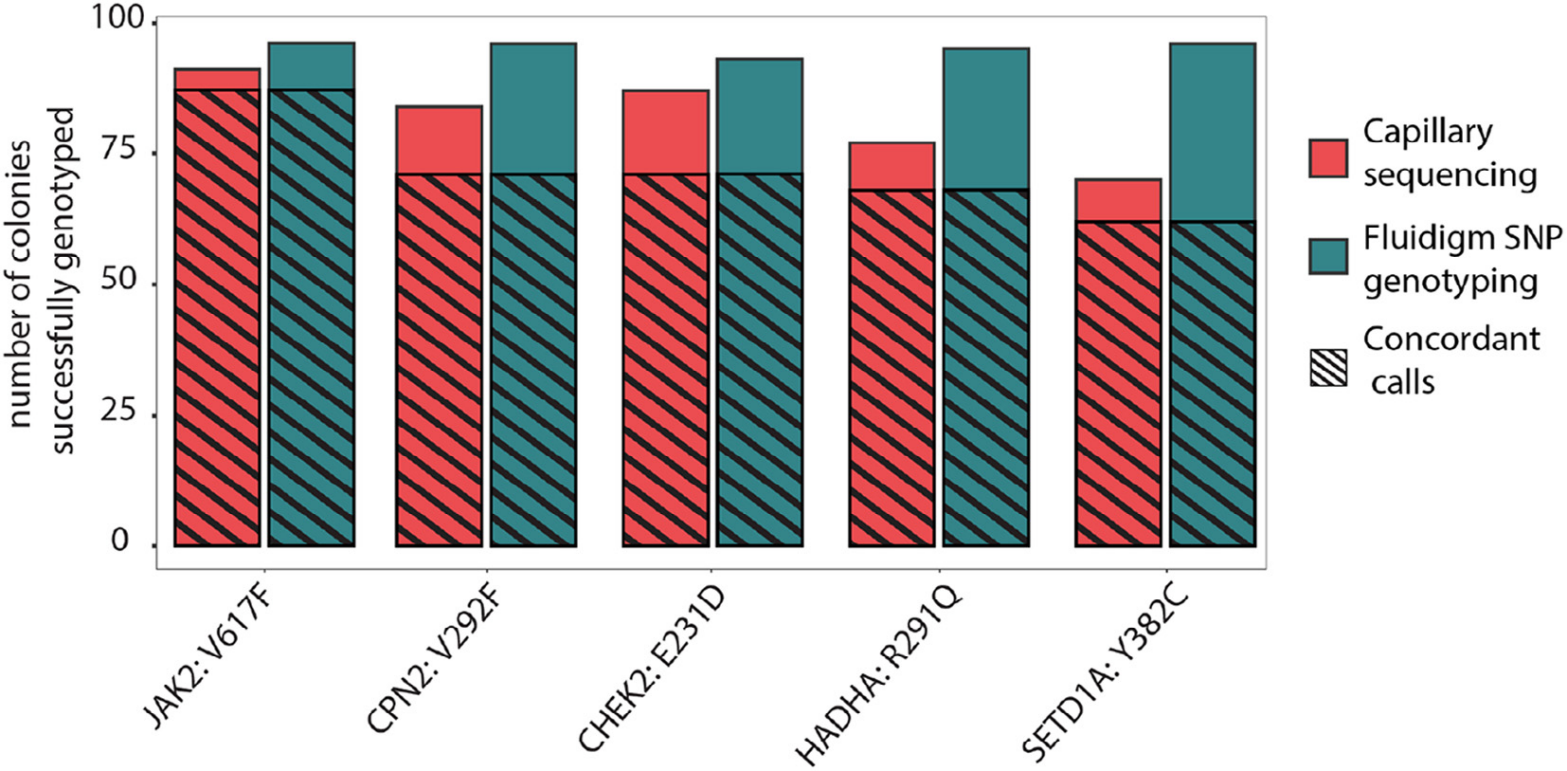
Multiplexed SNP genotyping is an efficient method for genotyping BFU-E colonies*.* Number of colonies for which a genotype could be called out of 96 colonies for each mutation is shown. Five genes were genotyped by capillary sequencing and multiplexed SNP genotyping. Shaded regions highlight the number of colonies for which the same genotype was called by both technologies.

To genotype simultaneously and efficiently a large number of BFU-E colonies, for all 16 mutations identified, we established a multiplexed assay based on custom SNP genotyping technology provided by Fluidigm. Firstly, we compared the performance of Fluidigm multiplexed SNP genotyping with classical capillary sequencing by genotyping a subset of 96 colonies for five mutations with both technologies. Fluidigm SNP genotyping returned a genotype in 476 of the 480 genotyping reactions (96 colonies × 5 mutations, an average call rate of 99.1%). In contrast, capillary sequencing resulted in a total call rate of 409/480 or 85.2% (Fig. 2). The genotyping results were then compared using only those colonies for which both methods yielded a genotype. On average, 87.7% of reactions returned concordant calls between the two methods (range 81.5–95.6%) (Fig. 2). Given the increased time efficiency with which multiplexed genotyping can be performed and the higher genotyping call rates, in addition to the considerable overlap in results compared with capillary sequencing, we decided to use multiplexed genotyping assays to genotype BFU-E colonies.

**Figure 3:**
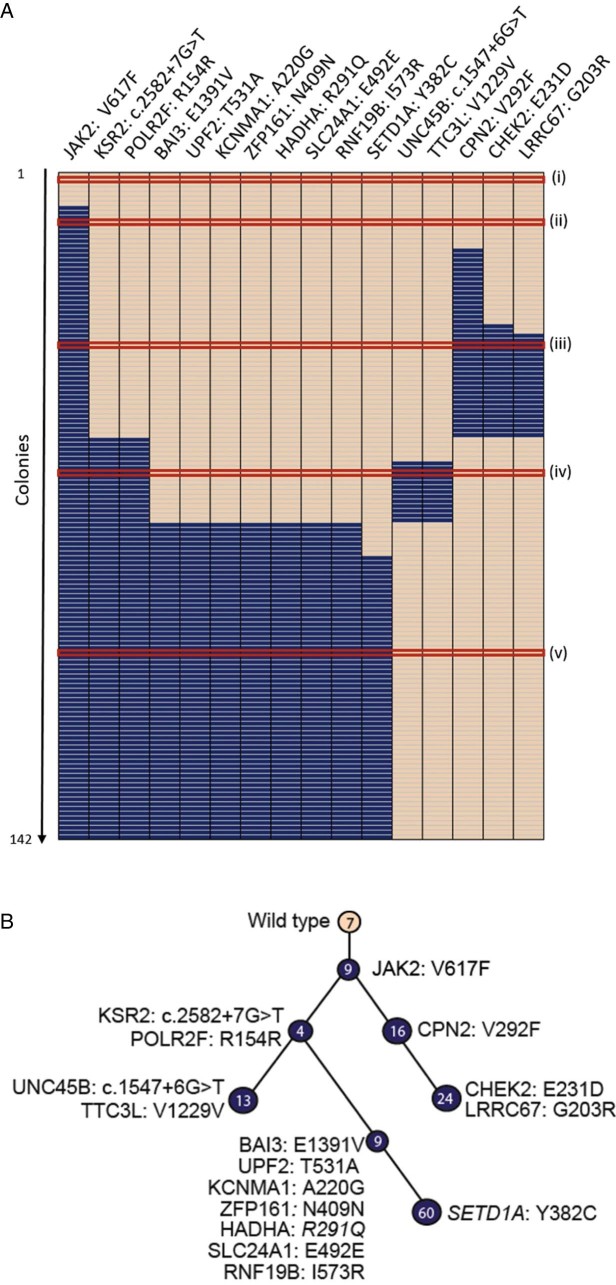
Schematic representation of the subclonal structure and evolution of the tumor for patient PD4772. Wild-type colonies with no mutations are shown in peach and heterozygous mutations are shown in blue. No colonies with homozygous mutations were identified in this patient. Each individual cluster of colonies representing a single subclone is highlighted by a Roman numeral and the number of colonies within the subclone. **(A)** Row-wise representation of the mutational status of each colony for each mutation. Each row is one colony and each column is one gene. Red lines delineate clusters of colonies with the same mutational characteristics. **(B)** Clonal hierarchy derived from the data in **(A)**. A node further down the hierarchy includes all mutations that precede it on the branch.

All 176 colonies from patient PD4772 were then genotyped for all 16 mutations using Fluidigm SNP genotyping. To best assess the order of mutation acquisition, the results were compiled in a tabular format to show the particular genotype (wild-type, heterozygous, or homozygous) for a specific mutation for a specific colony (Fig. 3A). The columns (genes) were ordered based on the frequency of the mutation in the 176 colonies so that the gene showing highest overall mutant allele burden is on the left. Colonies in rows are ordered to generate clusters of colonies with the same genotype (Fig. 3, nodes i–viii). The clusters represent genetically defined subclones of the tumor and the number of colonies within each cluster reflects the relative size of the respective subclone. Two colonies of a similar genotype were required as a minimum to call a separate cluster. When only one colony showed a certain mutational profile (which occurred in 34 of the 176 genotyped), the colony was removed from the analysis. Finally, all of the clusters were reordered so that the order of acquisition of subclones was readily visible.

These data were then converted into a clonal hierarchy (Fig. 3B). Within the hierarchy, each node represents a genetically defined subclone. Lines between nodes reflect the evolutionary relationship of subclones so that a more recently established subclone is connected to the clone from which it arose. Genetically, such a subclone carries all of the mutations of the parental clone and any newly acquired mutations.

Our results demonstrate that there is an unrecognized complexity to the subclonal architecture of the patient's tumor clone. After the initial mutation acquisition, JAK2^V617F^ (Fig. 3B, node ii), two independent subclones (Fig. 3B, nodes iii and v), which were identified by two distinct subsets of mutations, arose from the same parental tumor clone with the JAK2^V617F^ mutation. Within node iii, additional mutations weare acquired sequentially (Fig. 3B, node iv). After the acquisition of KSR2^c.2582+7G>T^, an additional bifurcation of the hierarchy occurred, with two sets of mutations acquired sequentially within the JAK2^V617F^/KSR2^c.2582+7G>T^ clone (vi and vii/viii).

Previous studies in MPNs have shown that mutational data from NGS alone can only be used to call mutation order in under half of cases 4, 5. In the case of low mutant allele burden, it is also not possible to tell whether mutations are acquired in a linear or biclonal fashion from NGS data alone. Here, we showed that combining NGS with highly multiplexed genotyping of BFU-E colonies is one method that can accurately determine the subclonal structure of a tumor. We could determine a highly complex subclonal structure, showing both the linear and biclonal acquisition of mutations, which was far more complex than the structure predicted from mutant allele burdens alone.

## Figures and Tables

**Table E1:** 

Gene	Protein Change	DNA Change	Chr	Position	NGS allele burden (%)
JAK2	p.V617F	c.1849G>T	9	5073770	46.9
CPN2	p.V292F	c.874G>T	3	194062558	21.3
HADHA	p.R291Q	c.872G>A	2	26437358	21.5
CHEK2	p.E231D	c.693A>T	22	29120993	16.3
SETD1A	p.Y382C	c.1145A>G	16	30976208	14.8
POLR2F	p.R154R	c.462G>T	22	38437084	23.6
KSR2	p.?	c.2582+7G>T	12	117914262	20.5
ZFP161	p.N409N	c.1227C>T	18	5290980	18.8
BAI3	p.E1391V	c.4172A>T	6	70071337	16.1
SLC24A1	p.E492E	c.1476G>A	15	65917894	14.7
RNF19B	p.I573R	c.1718T>G	1	33404025	13.3
UPF2	p.T531A	c.1591A>G	10	12043738	13
KCNMA1	p.A220G	c.659C>G	10	78944618	10.7
LRRC67	p.G203R	c.607G>A	8	67900698	10.5
TTC3L	p.V1299V	c.3897T>C	21	38538413	7.1
UNC45B	p.?	c.1547+6G>T	17	33496956	5.4

**Mutations, mutation locations and allele burdens for all mutations found by exome sequencing for patient PD4772**[1]. p., protein; p.?, splice site mutation; c., cDNA; Chr, chromosome; NGS, next generation sequencing. Genomic coordinates are from the hg19 reference genome.

**Table E2:** 

Gene	Protein Change	DNA Change	Chr	Position	Forward Primer	Reverse Primer
JAK2	p.V617F	c.1849G>T	9	5073770	CAAGCAGCA AGTATGAT GAGCAAGC	CTGACACC TAGCTGTG ATCCTGAA
CPN2	p.V292F	c.874G>T	3	194062558	TGGGAGGT GGGTAATG GCATTGTA	TCCATCTT TGCCTCCC TGGGTAAT
HADHA	p.R291Q	c.872G>A	2	26437358	TGGTCCAG AATGGCAA TAAGGAGGA	ACAGAATT GACAGCGTA TGCCATGA
CHEK2	p.E231D	c.693A>T	22	29120993	CACGCCCA GCAACTTA CTCATCTT	GAAGATCA CAGTGGCAA TGGAACC
SETD1A	p.Y382C	c.1145A>G	16	30976208	CTCCTCAT TGTCCTCG TCCTCCTC	AGGAGGTG TAAGAAGGT GGGAAGC

**Mutation locations and primer details used for capillary sequencing.** p., Protein; c., cDNA; Chr, chromosome. Genomic coordinates are from the hg19 reference genome.
